# Serotonergic Regulation in Alzheimer’s Disease

**DOI:** 10.3390/ijms26115218

**Published:** 2025-05-29

**Authors:** Lyudmila P. Dolgacheva, Valery P. Zinchenko, Alexander D. Nadeev, Nikolay V. Goncharov

**Affiliations:** 1 Federal Research Center “Pushchino Scientific Center for Biological Research of the Russian Academy of Sciences”, Institute of Cell Biophysics of the Russian Academy of Sciences, Pushchino 142290, Russiavpz@mail.ru (V.P.Z.);; 2 Sechenov Institute of Evolutionary Physiology and Biochemistry of the Russian Academy of Sciences, Saint Petersburg 194223, Russia

**Keywords:** Alzheimer’s disease, amyloid β oligomer, serotonin, serotonin receptors, mitochondrial dysfunction, mitochondrial biogenesis, neurogenesis, multitarget molecules

## Abstract

Serotonin (5-HT) is a neurotransmitter that also plays an important role in the regulation of vascular tone and angiogenesis. This review focuses on the involvement of the 5-HT system in pathological processes leading to the development of Alzheimer’s disease (AD). There is evidence that damage or dysfunction of the 5-HT system contributes to the development of AD, and different subtypes of 5-HT receptors are a potential target for the treatment of AD. A link has been established between AD, depression, stress, and 5-HT deficiency in the brain. There are new data on the role of circadian rhythms in modulating stress, depression, and the 5-HT system; amyloid β (Aβ) plaque clearance; and AD progression. Circadian disruption inhibits Aβ plaque clearance and modulates AD progression. The properties and functions of 5-HT, its receptors, and serotonergic neurons are presented. Special attention is paid to the central role of 5-HT in brain development, including neurite outgrowth, regulation of somatic morphology, motility, synaptogenesis, control of dendritic spine shape and density, neuronal plasticity determining its role in network regeneration, and changes in innervation after brain damage. The results of different studies indicate that the interaction of amyloid β oligomers (AβO) with mitochondria is a sufficient trigger for AD-related neurodegeneration. The action of 5-HT leads to an improvement in mitochondrial quality and the restoration of brain areas after traumatic brain injury, chronic stress, or developmental disorders in AD. The role of a healthy lifestyle and drugs acting on serotonin receptors in the prevention and treatment of AD is discussed.

## 1. Introduction

Recent studies increasingly point to the involvement of serotoninergic neurons in the pathological processes underlying AD. Damage to these neurons is associated with the deterioration of cognitive processes not only in aging and AD, but also in other mental disorders, including schizophrenia, stress, and depression [1,2,3].

The 5-HT system is multifaceted, affecting general mechanisms of AD and other neuropathologies, as well as specific mechanisms unique to AD. For example, the 5-HT system is involved in the control of neuroinflammation, oxidative stress, and mitochondrial dysfunction, which contribute to the development of a wide range of neuropathologies [4,5,6,7,8].

On the other hand, a growing body of data demonstrates the involvement of the 5-HT system in the mechanisms of formation of insoluble aggregates of Aβ and (hyper)phosphorylated tau protein (pTau), which are the main histopathological signs of Alzheimer’s disease [9]. This disorder is characterized by the accumulation of two abnormally folded proteins: β-amyloid (Aβ), forming extracellular senile plaques, and pTau, forming intracellular tangles. Different subtypes of 5-HT receptors and the intracellular signaling cascades they trigger are involved in various neuropathologies. Therefore, agonists and antagonists of different 5-HT receptors are being actively studied both as antidepressants and antipsychotics for the treatment of behavioral symptoms of different neuropathologies [10], and as drugs that affect specific causes of AD, namely the accumulation of Aβ and pTau [11]. Dysfunction of the 5-HT system occurs in the development of AD [1], but the mechanism of dysfunction is not fully understood. The number of 5-HT neurons in the dorsal raphe nuclei, the levels of 5-HT and its metabolite 5-hydroxyindoleacetic acid, and the binding strength for 5-HT in the cortex and hippocampus are significantly reduced in AD [12].

Selective 5-HT reuptake inhibitors, including escitalopram, citalopram, and fluoxetine, have beneficial effects on both psychiatric symptoms and, to a lesser extent, cognitive impairment in patients with AD [13,14,15]. Serotonin-selective antidepressants significantly improve depressive symptoms and daily functioning in patients with AD and are used to treat aggressive behavior. By modulating both the proliferation and survival of newly formed cells, serotonin is a key regulator of adult neurogenesis [16].

## 2. Stress, Depression, and the Development of AD

Alzheimer’s disease is a chronic neurodegenerative disorder that occurs as a result of the combined action of a number of factors: genetic disorders, aging processes, and accumulation of protein aggregates. AD causes progressive dysfunction and death of neurons in the cerebral cortex and certain subcortical areas associated with cognitive abilities and memory [17]. In AD, neurons associated with memory in the cortex and hippocampus are initially destroyed [18]. Later, areas of the cerebral cortex responsible for spatial navigation, language, thinking, and social behavior are affected.

AD occurs in two forms: familial (hereditary) and sporadic. In the hereditary form, genetic mutations are observed in the presenilin 1 and 2 (*PSEN1* and *PSEN2*) and amyloid precursor protein (*APP*) genes. Mutations in these genes increase Aβ production, raise the Aβ42/Aβ40 ratio, and promote plaque formation [19]. The combined effect of unhealthy lifestyle, environment, and genetic factors causes the sporadic form of the disease.

The most common manifestations of AD are short-term memory impairment and impaired expressive speech. Morphologically, AD is characterized by the formation of extracellular amyloid plaques, consisting mainly of fibrillar Aβ, as well as intracellular neurofibrillary tangles (NFTs), consisting of pTau. These two toxic protein compounds, Aβ and pTau, come into play at different time stages of the disease [20,21]. Aβ accumulation has been shown to be a marker of early Alzheimer’s disease, and pTau is an indicator of late stages of the disease [22,23]. Both Aβ and pTau affect the electron transport chain (ETC), which affects energy production [24,25], and also induce mitophagy, which results in excessive loss of mitochondria [26,27].

The Aβ protein is a central component of extracellular amyloid plaques in AD. Aβ causes mitochondrial dysfunction by inhibiting mitochondrial transmembrane translocase, interacting with cyclophilin D to interfere with energy production, and promoting opening of the mitochondrial highly permeable pore (mPTP) [25,28]. Aβ peptides are produced by the enzymatic cleavage of APP [29]. APP is a transmembrane glycoprotein expressed largely within the central nervous system (CNS). It is known that APP plays a significant role in neural growth and maturation during brain development. Full-length APP is incorporated not only into the plasma membrane, but also into the membranes of intracellular organelles, where it can be converted via the non-amyloidogenic or amyloidogenic pathway. Under physiological conditions (non-amyloidogenic pathways), APP is primarily first cleaved by α-secretase, producing a secreted form of APP (sAPPα) and a membrane-bound C-terminal fragment of 83 amino acids (CTF, C8), preventing Aβ production [30]. The non-amyloidogenic pathway plays a significant role in maintaining neuronal homeostasis, in signaling and intracellular transport, and is also involved in synaptogenesis and synaptic plasticity [31]. The non-amyloidogenic pathway is the most predominant proteolytic processing of the APP in healthy brain [32]. The secreted form of sAPPα exerts beneficial physiological, biochemical, and behavioral effects by counteracting the detrimental effects of neurotoxic agents. It has been shown to stimulate neurite outgrowth in neurons, promote clearance of Aβ from damaged microglia by restoring mitochondrial function, and reverse age-related declines in neural progenitor cell proliferation [33,34,35].

Amyloidogenic proteolytic processing of APP on the cell surface leads to the formation of Aβ [36,37]. Aβ oligomers (AβOs) alter the morphology and density of synapses and disrupt synaptic plasticity. Impaired synaptic function is considered an early and key pathology of AD. The most common Aβ species are 27–43 amino acids in length. Once produced, these short Aβ isoforms are secreted into the extracellular space as monomers via exosomes [38]. Long intracellular Aβ (iAβ) isoforms, such as Aβ45, aggregate in neurons [39,40]. Phosphorylation also causes protein aggregation and insoluble protein formation. iAβ accumulation is associated with the glymphatic system, particularly the paravascular pathway, which plays a key role in the clearance of extracellular Aβ mediated by astroglial aquaporin (AQP4) [19]. The release of Aβ fragments first leads to the spontaneous aggregation of Aβ monomers into soluble AβO, which is promoted by the presence of hydrophobic amino acids. The further aggregation of oligomers results in the formation of insoluble protofibrils [41].

AβO can exert toxic effects by directly interacting with pTau, lipid, and cholesterol components of the cell membrane, leading to the formation of channels and the destruction of membrane integrity and permeability. The latter allows Ca^2+^ ions to enter the cell, inhibit long-term potentiation (LTP), and induce neuronal death [42,43]. In addition, AβO contributes to other pathological manifestations of AD, including neuroinflammation, oxidative stress, and mitochondrial dysfunction, which also lead to neuronal death. Aβ levels are also modulated by the sleep–wake cycle [44]. A higher production and release of Aβ into the extracellular space has been observed during wakefulness, while enhanced Aβ clearance via the glymphatic system has been documented during sleep [45].

Stress and depression make a significant contribution to the development of AD [46,47]. Symptoms of depression are numerous and varied. They include apathy, feelings of guilt, worthlessness, hopelessness over a long period of time, sleep problems, and inability to concentrate [48,49,50]. Depression is thought to accelerate neurodegenerative processes, as individuals with depression are more likely to develop AD [51]. AβO increases long-term depression (LTD) in the hippocampus. Clinical data indicate that in both pathologies, atrophic changes occur in the same areas of the brain—the hippocampus and prefrontal cortex (PFC). These brain regions play a key role in regulating the stress response. Thus, depression may be an initial sign of neurodegenerative disease and may be considered a risk factor for the further development of AD [47].

Proposing that a deficiency of brain monoamines, including 5-HT, triggers the onset of depression, the monoamine–serotonin hypothesis of depression was proposed in the 1960s [52,53,54]. Acute depletion of tryptophan (a precursor of serotonin) causes the relapse of depression in patients, and the administration of 5-HT antidepressants causes remission [55,56,57]. Selective serotonin reuptake inhibitors (SSRIs) are common protective drugs against LTD [58].

The circadian and stress systems are also closely interconnected. Normally, the stress system is under circadian control and optimizes interactions with the environment [59]. In turn, the stress system can have feedback with the circadian system and provide information about time to the entire organism [59]. People with depression are more likely to have changes in their circadian rhythms, such as sleep–wake cycles, body temperature, neurotransmitter levels, and hormones [60]. In mammals, the circadian system is controlled by a central pacemaker known as the suprachiasmatic nucleus (SCN) of the hypothalamus [61]. The circadian system not only indirectly but also directly controls the development of AD. In experimental models of AD in mice, intact circadian rhythms promoted the removal of Aβ plaques, whereas the disruption of circadian rhythms inhibited their removal [62,63].

At the same time, the circadian system is closely linked to the stress-sensitive serotonin system [64]. There is a bidirectional relationship between 5-HT and the circadian system. Serotonergic antidepressant drugs treat the consequences of circadian rhythm disruption and, conversely, the 5-HT system is under circadian control. At the genetic level, key signaling molecules of the serotonin signaling network, such as the Na^+^/Cl^−^-dependent serotonin transporter (SERT) and the 5-HT_1B_, 5-HT_7_, and 5-HT_2C_ receptors, are expressed in the biological clock core SCN [65,66,67,68], where they mediate the effects of serotonin on rhythms. Early-morning light exposure [69], daytime exercise [70], and time-restricted diets [71,72] are all helpful in combating symptoms of depression, protecting against memory loss, and promoting longevity.

The activity of the glymphatic system, which regulates amyloid clearance from the brain via the perivascular space surrounding blood vessels, is also regulated by circadian rhythms. Dysfunction of the glymphatic system also plays a vital role in the severity of AD. The activity of this system is higher during sleep and lower during wakefulness [73,74]. In addition to local protein degradation mechanisms, plaque clearance via the glymphatic system is inhibited in AD when circadian rhythms are dysregulated. Acute sleep deprivation increases Aβ accumulation in humans [75].

## 3. AβO, 5-HT, and Mitochondrial Dysfunction

Mitochondria are essential organelles that provide neuronal functioning through their part in energy production, calcium transport, maintenance of redox balance, and programmed cell death [76]. Mitochondria are one of the main sources of reactive oxygen species (ROS). High levels of ROS can impair cell integrity through oxidation of lipids, proteins, and DNA. Many neurodegenerative diseases are accompanied by mitochondrial dysfunction [77,78].

AβO is considered to be the main cause of toxic effects in the AD brain [79,80]. The physical damage is partially attributable to the pore-forming effect of AβO [81]. AβO can incorporate directly into lipid bilayers, changing the structure of the cell membrane and thereby altering its permeability [82]. The barrel-like structure of AβO facilitates the formation of ion channels within the cell membrane, thereby enabling Ca^2+^ entry into the cell. It has been established that aggregated Aβ42 has the capacity to form ion channels, a property that is absent in Aβ40 [81]. It is hypothesized that another potential cause of mechanical damage to neurons is the interaction between AβO and lipid rafts [83].

A substantial number of reports have identified various forms of AβO receptors, as well as the associated signaling proteins. Well-studied AβO receptors include the cellular prion protein (PrPc) [84] and the neuron-specific Na/K-ATPase α3 subunit (NKAα3), which can promote neurodegeneration through presynaptic calcium overload [85]. PrPc binds to AβO, reducing the NMDA receptors’ (NMDARs’) density at synapses and leading to a loss of dendritic spines [86]. PrPc also forms complexes with AβO, activating metabotropic glutamate receptor 5 and inducing Ca^2+^ influx. Several other receptors have also been reported to bind directly or indirectly to AβO, thereby affecting neuronal survival.

In addition, several receptors have been reported to bind to AβO, allowing them to enter neurons in an endocytic manner. Aβ accumulates in lysosomal vesicles, which have a low internal pH. The acidic environment within endosomes and lysosomes serves as a preferential site for AβO formation [87]. The rate of assembly of AβO increases 8000-fold when they migrate from the neutral extracellular medium to the low-pH lysosomal environment [88]. Selective antibodies to AβO prevent neuropathology in a mouse model of AD and restore cognitive impairment in mice for at least 40 days [80,89,90]. Numerous data indicate that AβO receptors are not only necessary but also sufficient triggers of AD-associated neurodegeneration.

Increasing evidence suggests that AβO impairs mitochondrial function by disrupting mitochondrial dynamics and cristae structure, reducing mitochondrial quantity and quality, causing defects in mDNA, and inhibiting the mitochondrial ETC [91,92,93,94,95,96]. The resulting mitochondrial dysfunction initiates cell death processes.

It is currently known that Aβ accumulation in mitochondria is carried out by translocases of the outer (TOMs) and inner membranes (TIMs) [97,98]. It was reported by Sirk et al. that the rates of entry of two endogenous nuclear-encoded mitochondrial proteins, mortalin (mtHsp70) and TOM20, into mitochondria decreased under conditions of sublethal Aβ_42_ exposure, suggesting that Aβ_42_ occupies the TOM complex, thereby impairing its mitochondrial entry [99]. Inhibition of TOM20, TOM70, and TOM40 led to reduced Aβ accumulation in mitochondria. The presence of AβO can lead to an imbalance in mitochondrial dynamics that is manifested by increased mitochondrial fission and fusion, and increased mitochondrial fragmentation [100].

**Figure 1:**
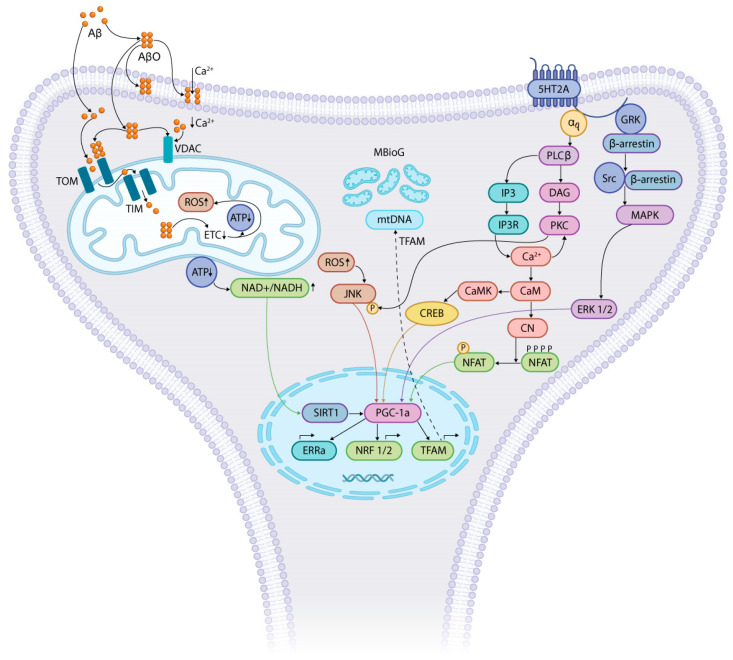
The barrel-like structure of AβO forms ion channels in the cell membrane, allowing Ca^2+^ to enter the cell. Ca^2+^ binds to VDAC, regulating its permeability and inhibiting ATP release into the cytosol. Aβ/AβO  enter the mitochondrial matrix (via TOM and TIM translocators localized on the outer and inner membranes) and interact directly with complex I of the respiratory chain, disrupting ETC function, decreasing ATP, and increasing ROS. The decrease in ATP leads to an increase in the (NAD^+^/NADH) ratio and activation of SIRT1. ROS production and the activation of JNK kinase are increased. SIRT1 and JNK penetrate the nucleus and activate PGC-1α. Serotonin regulation of mitochondrial biogenesis and function may be mediated by the 5-HT_2A_ receptor and PGC-1α, the master modulator of mitochondrial biogenesis. Activation of the 5-HT_2A_ receptor on neurons is accompanied by the stimulation of PGC-1α through several signaling pathways: (1) α_q_—PLC_β_—IP_3_—IP_3_R—↑Ca^2+^—↑CaM—↑CN—NFAT; (2) PLC_β_—DAG—PKC—JNK; (3) GRK—β-arrestin—β-arrestin/Src—MAPK—ERK1/2; (4) α_q_—PLC_β_—IP_3_—IP_3_R—↑Ca^2+^—CaM—↑CaMK—CREB. Abbreviations: Aβ—amyloid-β; AβO—Aβ oligomer; α_q_—subunit of heterotrimer protein G_q_; ATP—adenosine triphosphate; CaM—calmodulin; CaMK—calmodulin kinase; CN—calcineurin; CREB—cAMP-response element-binding protein; DAG—diacylglycerol; ERRa—estrogen-related receptor alpha; ERK—extracellular signal regulated kinase; ETC—electron transport chain; GRK—G-protein-coupled receptor kinases; 5-HT_2A_—serotonin G-protein-coupled receptor, subtype 2A; IP_3_—inositol trisphosphate; IP_3_R—receptor of inositol trisphosphate; mtDNA—mitochondrial DNA; JNK—Jun amino-terminal kinase; MAPK—mitogen-activated protein kinase; MBioG—mitochondrial biogenesis; NFAT—nuclear factor of activated T cells; NRF1/2—nuclear respiratory factors 1/2; PGC-1α—peroxisome proliferator-activated receptor gamma coactivator 1-alpha; PLCβ—phospholipase C β; PKC—protein kinase C; ROS—reactive oxygen species; SIRT1—NAD^+^-dependent deacetylase sirtuin-1; Src—non-receptor tyrosine kinase; TIM—inner-membrane translocase; TOM—outer-membrane translocase; TFAM—transcription factor A, mitochondrial; VDAC—voltage-dependent anion channel.

In neurons, AβO induces mitochondrial dysfunction and inhibits mitochondrial autophagy, leading to the release of mitochondrial contents, which act as the top sensors of PANoptosis (apoptosis, necroptosis, and pyroptosis) [100]. ETC damage by AβO is the main cause of decreased oxidative phosphorylation. In addition to Aβ and pTau accumulation, AD pathogenesis in mitochondria is associated with increased mDNA damage, low synaptic ATP, increased oxidative stress, and defective autophagy and mitophagy. Dysfunctional mitochondria accelerate the pathological process by increasing phosphorylation of tau proteins, generating excessive amounts of ROS, and activating pathways that lead to cell death [77]. Serotonin plays an important role in the regulation/restoration of synaptic transmission of neurons damaged in AD by improving mitochondrial function (Figure 1).

## 4. Metabolism and Functions of Serotonin in the CNS

Serotonergic neurons are highly branched, with axons reaching all areas of the brain. Serotonin regulates many physiological processes in neuropathologies, including neuronal stability, energy homeostasis, circadian rhythm, and neurogenesis [101,102]. By increasing neurogenesis in the hippocampus, 5-HT may exert an antidepressant effect [101,102].

Serotonin is synthesized from the amino acid L-tryptophan and is unable to penetrate the CNS through the blood–brain barrier [103]. L-tryptophan from the gut enters the bloodstream, binds to blood albumin, and is transported across the blood–brain barrier to the CNS by a specific neutral amino acid carrier [104]. 5-HT synthesis in the CNS occurs in serotonergic neurons that are located in two relatively small dorsal and median raphe nuclei (DRN and MRN) of the reticular formation of the brainstem [105]. The synthesis of 5-HT is a two-step process. The first step, catalyzed by the rate-limiting enzyme tryptophan hydroxylase 2 (Tph2), is the oxidation of tryptophan to 5-hydroxytryptophan (5-HTP) [106]. The second step is the conversion of 5-HTP into serotonin (5-hydroxytryptamine, 5-HT) by aromatic L-amino acid decarboxylase. Serotonin is an antioxidant that inhibits the generation of ROS, malondialdehyde and carbonyls, prevents thiol oxidation, reduces the degradation of 2-deoxy-D-ribose, and prevents apoptosis [107]. The important role of 5-HT as a regulator of mitochondrial biogenesis and function has been demonstrated in rodent cortical neurons: 5-HT increased mitochondrial biogenesis, as reflected by increased mtDNA levels and mitochondrial gene expression [108]. This was accompanied by increases in cellular ATP, basal and maximal respiration, and reserve respiratory capacity. These mitochondrial effects play important roles in cell survival, neuronal plasticity, stress adaptation, and the regulation of aging [108].

**Figure 2:**
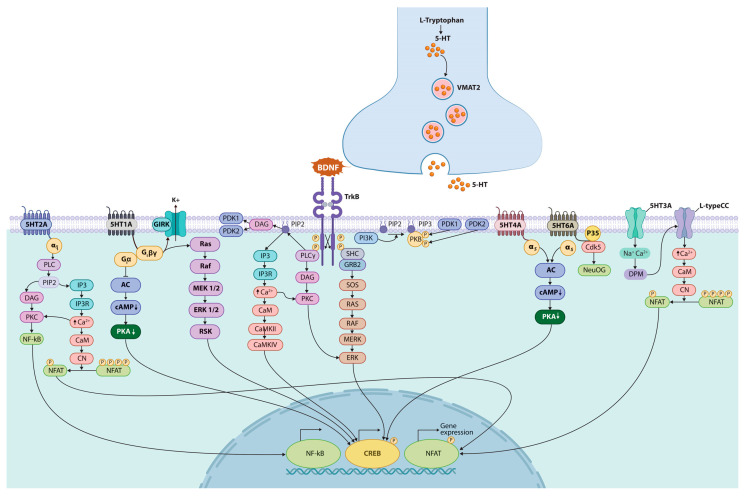
Serotonin and BDNF initiate neurogenesis. Adult hippocampal neurogenesis (AHN) persists throughout life in mammals, including humans. AHN is the creation (from neural stem cells (NSCs)) of new neurons, which integrate into the existing neural circuit of the adult brain. Most NSCs are concentrated in the dentate gyrus of the hippocampus and the subventricular zone (SVZ). The dentate gyrus of the hippocampus is one of the brain regions affected first in patients with Alzheimer’s disease. Neurogenic processes, including cell proliferation, differentiation, migration and maturation, are led by neurotrophic factors. Expression of BDNF in serotoninergic neurons increases stress tolerance and stimulates hippocampal neurogenesis in adults. BDNF activates the TrKB receptor, which acts via protein kinase C to activate proteins involved in cell survival and cell migration. The hippocampus expresses various 5-HT receptor subtypes, most of which (i.e., 5-HT1A, 2A, 3, 4, and 6) are expressed in the DG subregion of the hippocampus. The 5-HT_2A_ receptor is the main subtype of serotonin receptors mediating its excitatory action. 5-HT_2A_ receptors are localized predominantly in the plasma membrane and also enter the cell under the action of beta-arrestin. This results in the activation of NF-kB and NFAT, and mitochondrial biogenesis. The 5-HT_1A_ receptor activates the CREB and K^+^ channel. Selective stimulation of 5-HT_1A_ receptors increases the number of proliferating cells. Activation of the 5-HT_3_ receptor leads to membrane depolarization, the opening of voltage-dependent Ca^2+^ channels, and activation of calcineurin phosphatase and the NFAT transcription factor. 5-HT_4_ initiates the expression of BDNF. The 5-HT_6_ receptor is involved in the initiation of neurite outgrowth (NeuOG) by the agonist independently activating the cyclin dependent kinase 5 (Cdk5)-Cdc42 signaling pathway. The 5-HT signaling pathway activates the CREB, which promotes the transcription of the BDNF gene. Abbreviations: AC—adenylate cyclase; α_q_—subunit of heterotrimer protein G_q_; BDNF—brain-derived neurotrophic factor; CaM—calmodulin; cAMP—cyclic adenosine monophosphate; CaMK—calmodulin kinase; CN—calcineurin; Cdk5—cyclin-dependent kinase; CREB—cAMP-response element-binding protein; DAG—diacylglycerol; DPM—plasma membrane depolarization; ERK—extracellular signal regulated kinase; GIRK—G-protein-coupled inwardly rectifying potassium channels; GRB2—adaptor proteins containing SH2 and SH3 domains; G_iα_—heterotrimer protein G_i_; 5-HT—5-hydroxytryptamine (serotonin); IP_3_—inositol trisphosphate; IP_3_R—receptor of inositol trisphosphate; L-type CC—L-type calcium channel; MEK1/2—dual-specificity kinase; MERK—protein kinase; NeuOGs—neurite outgrows; NFAT—nuclear factor of activated T cells; NF-kB—nuclear factor kappa-light-chain-enhancer of activated B cells; p35—universal caspase inhibitor; PDK1 or 2—phospholipid-dependent kinase 1 or 2; PIP_2_—phosphatidylinositol diphosphate; PI_3_K—phosphatidylinositol 3 kinase; PLC_γ_—phospholipase Cγ; PKA—protein kinase A; PKB—protein kinase B; PKC—protein kinase C; PM—phosphatase; Raf—serine/threonine kinase; Ras—small G-protein, GTP-ase; RSK—ribosomal S6 kinase; SHC—Src homology 2 domain containing transforming proteins; SOS—guanine nucleotide exchange factor; TrKB—tropomyosin receptor kinase B; VMAT2—vesicular monoamine transporter 2.

Serotonin plays two key roles. First, during early development, it acts as a growth factor, regulating the development of the nervous system [109,110]. 5-HT is involved in many aspects of neuronal development, including neurite outgrowth, synaptogenesis, control of dendritic spine shape and density, and the development and function of key stress response systems [111,112,113]. In doing so, the 5-HT system interacts with the brain-derived neurotrophic factor (BDNF), S100beta, and other chemical messengers (Figure 2). As a developmental trophic factor, 5-HT regulates a variety of important processes, such as cell division, survival, differentiation, migration, myelination, synaptogenesis, and dendrite pruning [109,114]. A unique property of the 5-HT system is the ability to restore and re-innervate brain areas after a traumatic brain injury and chronic stress [115,116,117]. By regulating neurite outgrowth and synaptogenesis, 5-HT exerts profound effects on neuronal plasticity in the developing and mature nervous system [118,119]. The resulting changes in early-life control have critical consequences for behavior and mental health throughout life [120]. The disruption of these developmental processes may contribute to CNS disorders.

Second, serotonin acts as a potent neuromodulator in the central nervous system across the entire spectrum of vertebrate and invertebrate eukaryotes. As a neurotransmitter, 5-HT plays a central role in brain development and the regulation of attention, pain, mood and emotion, sleep, and arousal. 5-HT also plays a crucial role in the development and function of key stress response systems (the hypothalamic–pituitary–adrenal (HPA) and locus coeruleus–norepinephrine (LC-NE) systems) [111,121]. Neurons of the 5-HT system modulate a wide range of functions, including perception, mood, appetite, aggression, and anxiety [105]. 5-HT is also associated with cognition, memory, learning, sexuality, attention [122], respiratory stability [123], sleep–wake cycles, and circadian rhythms [124]. The serotonergic system also has a modulating effect on some hormones and neurotransmitters, such as dopamine, adrenaline and γ-aminobutyric acid (GABA), cortisol, prolactin, acetylcholine, oxytocin, substance P, and vasopressin [125,126,127]. Neural circuits capable of generating rhythmic behavioral actions, such as breathing, are modulated by the activity of serotonin receptors [128]. The anterior cingulate cortex (ACC) is a key brain region responsible for processing pain information and is under dense serotonergic innervation. The ACC is responsible for a variety of cognitive functions, including emotional expression, attention, and mood regulation.

Imbalances in serotonergic signaling are observed in many pathophysiological conditions, including attention deficit hyperactivity disorder, anxiety disorders, major depressive disorder (MDD), and AD [129,130]. Aβ deposits at the projection sites of serotonergic neurons may cause the retrograde degeneration of these neurons. Serotonin deficiency may be responsible for the increase in aggressive behavior and depression often observed in patients with AD. In several mouse models of AD-like brain amyloidosis, a decrease in the number of serotonin-producing neurons in the raphe nucleus and a decrease in the density of serotonergic fibers in several brain regions, including the neocortex and hippocampus, have been found [7]. SSRIs are common first-line treatments for major depression [54]. Acute depletion of tryptophan (a precursor of serotonin) causes a relapse of mild depressive symptoms in patients who had remitted with 5-HT antidepressants [55,56,57].

The functional effects of 5-HT are inhibited by the SERT of the neurotransmitter–sodium–symporter family [131]. In 5-HT neurons, SERT is expressed near the sites of serotonin release. By the reuptake of serotonin into presynaptic neurons, SERT limits the magnitude and duration of the 5-HT signal. As a result, SERT is a major target of antidepressants. Binding of inhibitors to the allosteric site of human SERT (hSERT) prevents the dissociation of antidepressants bound at the central site and may enhance the efficacy of such antidepressants, potentially reducing their dosage and side effects. In addition to SERT, other transporters are expressed in the brain that can mediate the diffusion of serotonin and other monoamines across cell membranes. These transporters, which include the organic cation transporters OCT1, OCT2, and OCT3, and the plasma membrane monoamine transporter PMAT, are less specific and can move catecholamines and histamine in addition to serotonin. Compared to SERT, they have a relatively low affinity for serotonin but have a significantly higher capacity [132]. Once released, serotonin exerts potent and diverse effects on neurons and other cells through a large family of 5-HT receptors [133] (Figure 2).

## 5. Serotonin Receptors

The actions of serotonin on target cells, including glutamate and GABA neurons, are mediated by seven receptor subtypes (5-HT_1–7_) in the central nervous system (CNS), peripheral nervous system (PNS), and blood vessel cells of the cardiovascular system (CVS) (Table 1). Most of them are G-coupled ones and only 5-HT_3_ receptors are ion channels permeable to sodium and potassium cations [134,135].

The 5-HT_1_R and 5-HT_5_R subtypes couple with the G_i/o_ protein, leading to the inhibition of adenylyl cyclase (AC) activity and a reduction in cyclic adenosine monophosphate (cAMP) levels [136]. 5-HT_2_R couples with the G_q/11_ protein, stimulating phospholipase C, which leads to elevated levels of inositol triphosphate and calcium ions [137]. The 5-HT_4_, 5-HT_6_, and 5-HT_7_ receptors couple with the G_s_ protein to enhance AC activity and elevate cAMP levels. Notably, 5-HT_4_R can bind to both G_s_ and G_i_ proteins with a preference for G_s_ over G_i_.

**Table 1:** Serotonin receptors, localization, agonists, and antagonists relationship to AD. References for general and signaling information [135,138,139,140,141,142,143,144,145,146,147,148,149,150,151,152,153,154,155]. Abbreviations for signaling: AC—adenylate cyclase; CAS—crk-associated substrate; DAG—diacylglycerol; eNOS—endothelial NO-synthase; ERK1/2—extracellular signal-regulated kinases 1 and 2; HMGB1—high-mobility group protein 1; IP_3_—inositol 1,4,5-trisphosphate; mTOR—mammalian target of rapamycin; NOX—NADPH oxidase; P38MAPK—p38 mitogen-activated protein kinase; PDK-1—3-phosphoinositide-dependent protein kinase-1; PIP_2_—phosphatidylinositol 4,5-bisphosphate; PKA—protein kinase A; PKB—protein kinase B (Akt); PKC—protein kinase C; PLC—phospholipase C; ROCK—rho-associated protein kinase; p70S6K1—ribosomal protein S6 kinase beta-1; ROS—reactive oxygen species. Abbreviations for localization: CNS—central nervous system; CVS—cardiovascular system; GI Tract—gastrointestinal tract; PNS—peripheral nervous system; SMC—smooth muscle cells. Chemical names for the research compounds without trivial names are listed in Appendix A.

Receptor *K*_d_ Subtype	Signaling	Agonists	Antagonists	Localization			AD Related [References]
				CNS	CVS	Other	
5-HT_1A_ 2.65 nM Metabotropic	G_i_/G_o_—AC—PKA G_i_/G_o_—PI_3_K—PKB—eNOS G_i_/G_o_—P38MAPK—HMGB1 G_i_/G_o—_NOX1/4—ROS—ROCK—ERK1/2	Xaliprofen Ipsapirone BP 554 8-OH-DPAT U92016A	WAY100635 NAN190	+	+		✓ [11]
5-HT_1B_ 16.01 nM Metabotropic	G_i_/G_o_—AC—PKA	CGS 12066B CP-93129 Ergotamine Eltoprazine Triptans (Zolmitriptan Sumatriptan, Eletriptan) Nonyloxytryptamine	GR-127935 GR55562 Isamoltane SB236057 NAS-181	+	+		✓ [156]
5-HT_1D_ 10.05 nM Metabotropic	G_i_/G_o_—AC—PKA	PNU-109291 L-703,664 GR 46611 Ergotamine Alniditan Triptans (Zolmitriptan, Sumatriptan, Eletriptan, Frovatriptan, Naratriptan, Almotriptan)	SB272183 LY310762 BRL15572 Cyanopindolol	+	+		✓ [157]
5-HT_1E_ 7.0 nM Metabotropic	G_i_/G_o_—AC—PKA	5-CT BRL54443		+	+		
5-HT_1F_ 67.60 nM Metabotropic	G_q/11_—PLC—PIP_2_—IP_3_—Ca^2+^—DAG G_q/11_—NOX—ROS—PI_3_K—PKB—mTOR—p70S6K1	LY334370 BRL54443 LY344864	BRL-54443 Lasmiditan		+		
5-HT_1P_ Metabotropic		5-Hydroxy-indalpine	5-HTP-DP			GI Tract	
5-HT_2A_ 970.80 nM Metabotropic	G_q/11_—PLC—PIP_2_—IP_3_—Ca^2+^—DAG G_q/11_—ERK1/2—eNOS	DOB DOI α-Methyl-5HT TCB-2	R-95544 Volinanserin Sarpogrelate	+	+	GI Tract, Platelets, PNS, SMC	✓ [11]
5-HT_2B_ 11.35 nM Metabotropic	G_q/11_—PLC—PIP_2_—IP_3_—Ca^2+^—DAG	BW723C86 DOB DOI	LY272015 RS127445	+	+	GI Tract, Platelets, PNS, SMC	✓ [158]
5-HT_2C_ 35.58 nM Metabotropic	G_q/11_—PLC—PIP_2_—IP_3_—Ca^2+^—DAG	WAY 163909 MK212 1-Methyl-psilocin DOB DOI	RS102221 SB242084	+	+	GI Tract, Platelets, PNS, SMC	✓ [159]
5-HT_3_ 190.33 nM Ionotropic		SR57227 2-Methyl-5HT Phenylbiguanide	MDL 72222 Tropisetron Ondansetron Granisetron	+		GI Tract, PNS	✓ [160]
5-HT_4_ 117.0 nM Metabotropic	G_s_—AC—PKA	RS67506 BIMU1 BIMU8 RS67333 Zacopride	GR113808 RS100235 SB204070	+		GI Tract, PNS	✓ [161]
5-HT_5A_ Metabotropic	G_i_/G_o_—AC—PKA		SB 699551	+			✓ [162]
5-HT_5B_ Metabotropic				Rodent CNS			
5-HT_6_ 116.53 нM Metabotropic	G_s_—AC-PKA Fyn—ras-MEK-ERK1/2	WAY 181,187 EMD 386088	Ro 04-6790 SB 399885 SB271046	+			✓ [163]
5-HT_7(a-d)_ 3.65 нM Metabotropic	G_s_—AC—PKA	LP12 LP44 AS-19 5-CT LP211	SB-269970 SB-258719	+	+	GI Tract	✓ [164]

### 5.1. 5-HT_1_ Receptors

The 5-HT_1_ receptor family includes 5-HT_1A_, 5-HT_1B_, 5-HT_1D_, 5-HT_1E_, 5-HT_1F_, and 5-HT_1P_ receptors that are associated with the Gi/Go protein, the α_i_ subunit of which inhibits AC activity and reduces cAMP levels. Most 5-HT_1_ receptors are postsynaptic, with the exception of 5-HT_1A_ and 5-HT_1B_, which are mainly presynaptic and modulate serotonin release. In serotonin neurons, 5-HT_1A_ receptors are located in the somatodendritic compartment [165]. In terminal areas of serotoninergic innervation, such as the hippocampus, the effect of 5-HT_1A_ receptors is realized through both α_i_ and βγ. However, in the dorsal raphe nucleus, 5-HT_1A_-receptors are associated only with βγ-dependent opening of potassium channels. Through Gβγ subunits, 5-HT_1A_ activation can induce the opening of inwardly rectifying potassium channels (GIRKs), which causes neuronal hyperpolarization [166,167,168]. It has been suggested that 5-HT_1A_ may also regulate not only GIRKs but also small-conductance Ca^2+^-activated potassium (SK) channels [169]. Most serotonergic neurons have a prominent medium-duration afterhyperpolarization (mAHP), which plays a critical role in setting the firing frequency by delaying the occurrence of the next action potential [170]. This mAHP is due to the opening of SK channels. 5-HT enhances mitochondrial axonal transport in hippocampal neurons via the 5-HT_1A_ receptor [171]. Administration of a 5-HT_1A_ receptor antagonist inhibits mitochondrial movement.

Thus, the ultimate effect of 5-HT_1A_ activation is a decrease in neuronal excitation rate and inhibition of protein kinase A. Activation of presynaptic 5-HT_1A_ receptors by 5-HT provides negative feedback and is considered a key mechanism of autoregulation of the brain 5-HT system [172,173]. Agonists of 5-HT_1A_, 5-HT_1B_, and 5-HT_1D_ receptors are being actively tested as therapeutic agents for AD [11,156,157].

### 5.2. 5-HT_2_ Receptors

The 5-HT_2_ receptor family includes the 5-HT_2A_, 5-HT_2B_, and 5-HT_2C_ receptors, the 5-HT_2A_ receptors being the most studied. This type of 5-HT receptor is conjugated to the Gq protein that stimulates PLC. PLC hydrolyzes the phospholipid PIP_2_ to IP_3_ and DAG, initiating Ca^2+^ ion elevation and PKC activation [137]. The activation of 5-HT_2A_ receptors mediates neuronal depolarization as a result of the closing of potassium channels (probably due to a decrease in PIP_2_, an agonist of these channels). The 5-HT_2A_ receptor is the main subtype of serotonin receptors mediating its excitatory effect and increase in neuronal excitability [174,175]. 5-HT_2A_ receptors are localized predominantly on the plasma membrane. However, a number of studies have provided evidence supporting their intracellular localization, for example, under the action of β-arrestin [174,176,177]. The activation of the 5-HT_2A_-β-arrestin signaling pathway has been shown to initiate an antipsychotic-like behavioral profile [178]. Furthermore, it has been suggested that the association of intracellular 5-HT_2A_ receptors with the microtubule-associated cytoskeletal protein MAP1A may be involved in intraneuronal signaling processes involved in cytoskeletal reorganization [179]. Serotonin regulation of mitochondrial biogenesis and function in rodent cortical neurons is mediated through the 5-HT_2A_ receptor and master modulators of mitochondrial biogenesis, SIRT1 and PGC-1α [108]. The effects of 5-HT on mtDNA, gene expression, ATP levels, and oxidative phosphorylation were observed upon the stimulation of the 5-HT_2A_ receptor of cortical neurons and utilized signaling pathways involving PLC and MAPK, but not PI3K-Akt. The 5-HT_2A_ receptors expressed on glutamatergic cortical pyramidal cells and deep cortical layers (V and VI) are involved in the regulation of behavioral responses to numerous psychotropic agents [180,181]. Serotoninergic neurotransmission mediated by 5-HT_2A_ receptors is a key target for therapy of sensory and cognitive disorders [182,183]. Interestingly, both 5-HT_2A_ and 5-HT_2B_ receptor antagonists [11,158] and 5-HT_2C_R [159] agonists have been used in attempts to correct AD-related conditions.

### 5.3. 5-HT_3_ Receptors

The 5-HT_3_ receptor is a ligand-gated ion channel. Homomeric and heteromeric 5-HT_3_ receptors mediate a rapidly activating, desensitizing, inward current, which is predominantly carried by sodium and potassium ions [184,185]. 5-HT_3_R is a homopentamer or heteropentamer and composed of five subunits, namely 5-HT_3_RA, 5-HT_3_RB, 5-HT_3_RC, 5-HT_3_RD, and 5- HT_3_RE [186,187]. The receptors are found both pre- and postsynaptically, and activation can modulate the release of a range of neurotransmitters, including dopamine, GABA, substance P, and acetylcholine [188,189,190].

Neurons in the neocortex, olfactory cortex, hippocampus, and amygdala that express the 5-HT_3_ receptor are primarily GABAergic neurons [191]. Approximately 50% of hippocampal interneurons express the 5-HT_3_ receptor [192,193]. In various animal models of AD, early Aβ plaques in the hippocampus are present exclusively in areas occupied by interneurons [194]. The 3A subunit of the 5-HT_3_ receptor was found to be significantly expressed in the brains of mice (AD model) and AD patients. In the mouse model, 5-HT_3_RA-positive interneurons were also clustered around Aβ plaques [195]. Treatment of mice with tropisetron, a 5-HT_3_R antagonist, for 8 weeks resulted in the partial reversal of cognitive deficits, and Aβ plaques and neuroinflammation were significantly reduced. These data led the authors to suggest that the inhibition of 5-HT_3_R GABAergic neurons at the initial stage of AD partially reverses the pathologic changes caused by the disease [195].

### 5.4. 5-HT_4_ Receptors

5-HT_4_R is an excitatory G_s_-coupled receptor that activates cAMP synthesis, the cAMP-PKA pathway, and increases neuronal excitability. Ten different splice variants (5-HT_4a_, 5-HT_4b_, 5-HT_4c_, 5-HT_4d_, 5-HT_4e_, 5-HT_4f_, 5-HT_4g_, 5-HT_4hb_, 5-HT_4i_, and 5-HT_4n_) have been found in humans [196]. 5-HT_4_R is widely expressed throughout the CNS and plays an important role in the regulation of mood, anxiety, and cognitive function, and drugs that activate this receptor have rapid antidepressant effects [196,197]. Loss of functional 5-HT_4_R in excitatory hippocampal neurons leads to persistent AD-like behavioral responses and increased anxiety [198]. 5-HT_4_R is required to maintain proper excitability of granular cells of the dentate gyrus (DG) [199,200]. 5-HT_4_ agonists may be used to treat cognitive impairment associated with depression. The activation of 5-HT_4_ receptors may improve cognitive symptoms due to their known ability to enhance acetylcholine release and to some extent compensate for the deficit in cholinergic transmission in AD [201]. In preclinical studies, short-term treatment with 5-HT_4_R agonists (RS67333 and others) had anxiolytic and antidepressant properties and mimicked the cellular and molecular responses of AD induced by chronic SSRI administration [199,200]. The 5-HT_4_ receptor has also been shown to be a target for the treatment of MDD, and the pharmacologic stimulation of these receptors has been shown to improve learning and memory in healthy subjects [202].

### 5.5. 5-HT_5_ Receptors

The 5-HT_5_ subtypes are metabotropic receptors that activate the Gi/Go protein, suppress AC activity, and reduce the level of cAMP. The 5-HT_5_ subfamily consists of two members, designated as 5-HT_5A_ and 5-HT_5B_. 5-HT_5A_ receptors are expressed in the cerebral cortex, hippocampus, and raphe nuclei [203]. They exhibit antinociceptive properties and are involved in the regulation of memory and learning [204]. Attempts have also been undertaken to use them as targets in the treatment of Huntington’s disease and AD [162].

### 5.6. 5-HT_6_ Receptor

Among the known serotonin receptor subtypes, 5-HT_6_R is the only one whose expression is restricted to the CNS. The 5-HT_6_ subtypes are metabotropic receptors that are conjugated to a Gs protein that activates AC and increases cAMP levels. The 5-HT_6_ receptor is known to not only activate AC but also interacts with Fyn kinase, mediating the Ras-MEK-ERK1/2 signaling pathway, and with c-Jun activation domain binding protein-1 (Jab1), which binds to the c-Jun transcription factor [205,206,207]. During development, 5-HT_6_ signaling regulates the migration of cortical pyramidal neurons and interneurons and morphogenesis of the dendritic tree [208,209]. In vivo studies on the developing cortex have identified the involvement of 5-HT_6_ receptors in dendritic growth and differentiation of neurons that transmit signals via the Fyn pathway [210,211].

5-HT_6_ receptors are expressed in different brain regions [212,213]. Studies on the localization of 5-HT_6_R in the hippocampus have shown that they are mainly expressed on excitatory pyramidal cells and to a much lesser extent on inhibitory GABAergic interneurons [214,215]. The application of 5-HT_6_ receptor antagonists increases the levels of acetylcholine and glutamate in the frontal cortex and hippocampus resulting in enhanced excitatory neurotransmission. 5-HT_6_ receptor antagonists inhibit the mTOR complex, which promotes neuronal survival and increases neurite outgrowth [216]. This modulation of the 5-HT_6_ complex by mTOR suggests a potential for the treatment of anxiety, schizophrenia, and AD [163].

In AD, a significant decrease in 5-HT_6_R density was found in the cortical regions of patients [217]. Preclinical studies on rodents and primates have reported that 5-HT_6_R antagonists improve cognitive function in a wide variety of learning and memory paradigms and account for the effects of antidepressants [218,219]. The absence of 5-HT_6_R receptors in peripheral organs makes it a preferable therapeutic target for treating the cognitive symptoms of schizophrenia, autism spectrum disorders, and dementia associated with AD. The efficacy of 5-HT_6_R antagonists in alleviating cognitive impairment has been demonstrated in a number of rodent models of neurodegenerative, psychiatric, and neurodegenerative diseases [220,221,222,223].

### 5.7. 5-HT_7_ Receptors

Different isoforms of 5-HT_7_R are expressed in humans: 5-HT_7_R(A), 5-HT_7_R(B), and 5-HT_7_R(D) [224]. The activation of 5-HT_7_ receptors initiates signaling not only through Gαs [225] but also activates RhoA and Cdc42 (the non-canonical signaling pathway of 5-HT_7_R acts via Gα12) [226]. This leads to the activation of Rho, Rac, and cell division control protein 42 (Cdc42); all of them are part of the Rho family of small GTPases, which in neurons promote dendrite sprouting, the formation of filopodia, and synaptogenesis [227,228,229,230]. Moreover, the 5-HT_7_ receptor can regulate serum response element (SRE)-mediated gene transcription as well as modulate the morphofunctional state of neurons through the Gα12-dependent activation of RhoA and Cdc42 GTPases [231]. A critical role of 5-HT_7_R in neuronal morphogenesis has been demonstrated: serotonin activation of 5-HT_7_R leads to increased dendritic spike density and enhanced synaptogenesis in forebrain neurons [232]. 5-HT_7_R is widely expressed in the CNS, the gastrointestinal tract, and other organs, where it potentially regulates various physiological functions, including the sleep–wake cycle, learning and memory, body temperature, nociception [233,234,235], and depressive-like behavior [236,237]. In the CNS, 5-HT_7_R is expressed in different cell types, including neurons, astrocytes, and microglia [226,238,239]. LP-211, a highly selective 5-HT_7_R agonist, ameliorated neuronal damage and cognitive impairment induced by Aβ [164]. In a streptozotocin-mediated murine model of AD neurodegeneration, intracerebroventricular treatment with the AS-19, 5-HT_7_R selective agonist reduced LTP impairment and apoptosis in the hippocampus [240]. In this way, 5-HT_7_R agonists may be neuroprotective by acting at multiple levels, including the reduction in excitotoxicity and oxidative stress, synaptic remodeling, regulation of neurotrophic factors, or immunomodulation.

## 6. Serotonin Level, Inflammation, and Immunity

As mentioned in Table 1, various 5-HT receptors are presented on the cells of the cardiovascular system, namely on smooth muscle and endothelial cells, but they also are presented on the various, at least 11, types of immune cells [241], leading to the serotonin level, inflammation, and immunity being interconnected in complex ways. Serotonin acts as a signaling molecule in both the central nervous system and peripheral immune system. Serotonin regulates both pro- and anti-inflammatory immune responses. Peripheral 5-HT regulates immune cell activity (e.g., T cells, macrophages, and dendritic cells).

Serotonin action on immune cells is multifaceted. 5-HT can enhance inflammation by activating Th1/Th17 cells and promoting the release of cytokines (e.g., TNF-α, IL-6) [242,243]. At other times, 5-HT may suppress inflammation by increasing the activity of regulatory T cells [244]. Gut-produced 5-HT influences systemic inflammation, which can affect brain depression [245]. Chronic inflammation from obesity or stress lowers 5-HT, which exacerbates mood disorders [246]. Reduced 5-HT levels have been found also in autoimmune diseases (e.g., rheumatoid arthritis, IBD) [247]. Pro-inflammatory cytokines (e.g., IL-1β, IL-6) can reduce serotonin levels [248] by reducing 5-HT precursor tryptophan via the kynurenine pathway (converting it to neurotoxic metabolites) [249]. Treatments targeting inflammation (e.g., anti-cytokine drugs) may help serotonin-related disorders. Chronic stress suppression reduces serum cortisol levels and inflammation, and increases 5-HT levels [250].

It should be noted that the inflammatory process in the CNS and the pathogenesis of AD are interrelated: the intensity of neuroinflammation increases as a result of neurodegeneration and Aβ deposition, which contributes to the strengthening of the same processes. As one of the mechanisms, it has been shown that an increase in the concentration of proinflammatory cytokines depresses melatonin synthesis in the pineal gland, which leads to the insufficiency of its effects, in particular, to the impaired regulation of circadian rhythms [251]. Furthermore, sleep deprivation leads to an increased production of proinflammatory cytokines, such as IL-1β, IL-6, and IL-17 [252]. As a result, a vicious circle between circadian rhythm regulation and neuroinflammation is formed.

## 7. Conclusions: Serotonin Is a Factor of Healthy Longevity

**Figure 3:**
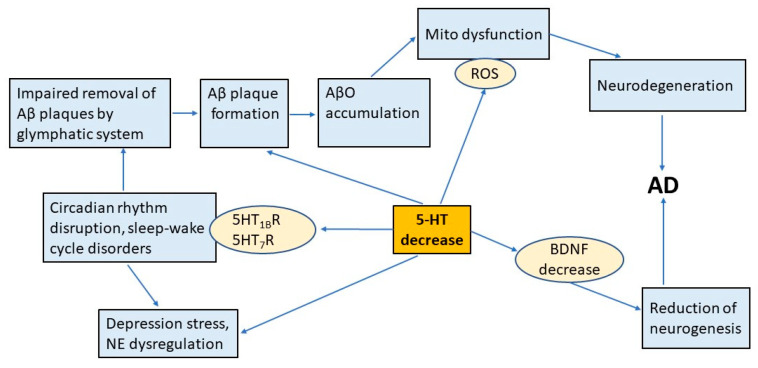
Relationships between serotonin, depression, neurodegeneration, AD, and the circadian clock system. 5-HT regulates circadian rhythms by acting via 5-HT_1_B and 5-HT_7_ receptors. Disruption of circadian rhythms can lead to sleep disturbances, depression, decreased BDNF, mitochondrial dysfunction, increased ROS, and oxidative stress. Depression impairs glymphatic system activity, promotes AβO formation and accumulation, and initiates AD. Abbreviations: Aβ—amyloid-β; AβO—β-amyloid oligomer; AD—Alzheimer’s disease; 5-HT—serotonin; BDNF—brain-derived neurotrophic factor; ROS—reactive oxygen species; NE—norepinephrine.

This review focuses on the involvement of the 5-HT system in the pathological processes leading to the development of AD. Figure 3 shows that the dysfunction of the 5-HT system causes AD progression through the disruption of circadian rhythms and the glymphatic system, Aβ plaque formation, mitochondrial dysfunction, neurodegeneration, decreased neurogenesis, depression, and stress. New data on the role of circadian rhythm disruption in glymphatic system dysfunction and Aβ accumulation in the brain, modulation of stress, depression, and the 5-HT system itself are discussed.

The 5-HT system remains a central therapeutic target in AD. Preclinical and clinical studies suggest that serotonergic dysfunction contributes to Aβ accumulation, neuroinflammation, tau pathology, and cognitive decline. Early loss of serotonergic neurons in the raphe nuclei precedes cognitive symptoms. The 5-HT system’s complexity and central role in multiple disorders make it a prime target for novel therapeutics, especially in mental health and neurology. Selective serotonin reuptake inhibitors (SSRIs) (e.g., citalopram, sertraline) remain first-line therapy but require dose adjustments due to altered pharmacokinetics in aging. Chronic SSRI use leads to the desensitization of these receptors, enhancing 5-HT release. SSRIs show slow Aβ accumulation in the brain [253] and decrease depression and anxiety. Anti-inflammatory agents (e.g., non-steroidal anti-inflammatory drugs and statins) may synergize with SSRIs.

Aging is associated with decreased 5-HT synthesis, reduced receptor density (e.g., 5-HT_1A_ and 5-HT_2A_), and impaired synaptic plasticity, all of which contribute to late-life depression (LLD). LLD increases the risk of AD via amyloid-β accumulation and the loss of 5-HT neurons in the raphe nuclei [12]. The activation of postsynaptic 5-HT_1A_ receptors promotes neurogenesis and resilience to stress, which are critical for LLD with cognitive impairment. 5-HT_4_ receptor agonists show promise in reducing amyloid pathology as well. Exercise and a Mediterranean diet increase 5-HT and BDNF levels, thereby improving mood and cognition. However, the role of the 5-HT system in LLD extends beyond monoamine deficiency and involves receptor dynamics, neuroinflammation, and vascular pathology, requiring tailored approaches.

The etiology and pathogenesis of neurodegenerative diseases, including AD, are multifactorial; therefore, one treatment strategy is to develop drugs that can act on multiple targets involved in disease pathogenesis. Increasing evidence suggests that enhancing the global antioxidant defense system may be more effective in controlling oxidative stress-related pathogenesis in the AD brain [254].

Serotonin levels are closely related to inflammation. On the one hand, 5-HT can suppress systemic inflammation, which affects brain depression [242,248]. On the other hand, chronic inflammation caused by obesity or stress reduces 5-HT levels, which exacerbates psychiatric disorders. Thus, treatments aimed at suppressing inflammation (e.g., anti-cytokine drugs) may help with 5-HT deficiency disorders. Anti-inflammatory diets (omega-3 fatty acids, polyphenols) can also improve 5-HT production and maintain 5-HT levels.

Hybrid polyfunctional molecules combining anticholinesterase activity and a high affinity for G-protein-coupled receptors appear promising for the treatment of AD. Thus, a series of such polyfunctional agents have been synthesized and tested: cholinesterase inhibitors possessing high selectivity to the type 3 histamine receptor [255], on cannabinoid type 2receptors [256,257] as well as on serotonin receptors: 5-HT_6_ [258] and 5-HT_4_ [259].

One of the first 5-HT_4_ receptor agonists of the benzimidazolone class is BIMU-8, which is able to cross the blood–brain barrier. It attenuates respiratory depression by activating the pre-Bötzinger complex located in the respiratory center of the brainstem [260]. The compound has also been shown to increase brain activity and improve learning and memory [261].

Multifunctional ligands are intended for use in both symptomatic and pathogenetic therapy. Nevertheless, the utilization of this approach is accompanied by a quantity of interrelated problems: (a) high molecular weight of “hybrid” molecules; (b) inability to cross the blood–brain barrier; (c) low bioavailability during oral administration; (d) the need to optimize selectivity and affinity for different targets; and (e) action of “hybrid” molecule components at different concentration ranges. It is essential to take into account these peculiarities at the very first stages to make appropriate computational studies before proceeding to the animal research. Recently, an algorithm has been proposed to design “functional binaries” that combine the properties of cholinesterase inhibitors and 5-HTR activators. At the first stage, a group of substituted 1,3-dihydro-2-oxy-1*H*-benzimidazol-2-ones was formed for further synthesis. Using Percepta software, logP, logBB, and LD_50_ (mg/kg) were theoretically calculated for mice and rats. Based on the data obtained, the most promising compounds were selected, which were subsequently synthesized and their anticholinesterase activity was investigated under in vitro conditions [262]. The biochemical analysis performed showed the ability of the obtained structures to inhibit AChE and BChE. In the next stage of the experimental research, the interaction of several substituted 1,3-dihydro-2-oxo-1*H*-benzimidazol-2-ones with three types of G_s_-protein-coupled serotonin receptors, 5-HT_6_, 5-HT_4_, and 5-HT_7_, was investigated in silico. Molecular modeling methods, such as molecular docking and molecular dynamics simulations in water and a lipid bilayer, were used to study the interaction of the compounds with 5-HTR. In addition, molecular modeling methods were used to investigate the mechanism of interaction of the tested compounds with cholinesterases to describe the binding sites, and to reveal the structural features of the drugs that determine the potency of their anticholinesterase activity. A primary in silico evaluation showed that benzimidazole–carboxamides effectively bind to 5-HT_4_R and 5-HT_7_R. The pool of the obtained data allows us to choose N-[2-(diethylamino)ethyl]-2-oxo-3-(tert-butyl)-2,3-dihydro-1*H*-benzimidazole-1-carboxamide hydrochloride as the most promising for further experimental development [263].

Physical exercise is known to have positive effects on brain function. For example, walking, especially high-intensity walking, is associated with improved episodic memory, even when started in middle age [264]. Exercise improves mood by enhancing mitochondrial function and neuroplasticity in the dorsal raphe [265]. Multicomponent training programs may be an important non-pharmacologic strategy to improve physical and cognitive function in hospitalized AD patients [266]. Short-term aggravated exercise reduced neuroinflammation and attenuated neuropathological changes in AD mice [267]. Combined multimodal exercise is superior to aerobic and weight-bearing exercise in terms of stimulating executive function [268]. Regular aerobic exercise can increase serotonin release and modulate synaptic plasticity in the anterior cingulate cortex, ultimately reducing pain and associated anxiety behaviors through the functions of serotonin 5-HT_1A_ and 5-HT_7_ receptors [267].

Increasing evidence indicates a strong association between a poor diet and the exacerbation of mood disorders, including anxiety and depression, as well as other neuropsychiatric conditions. The current epidemiological data on nutrition and mental health do not provide information about causality or underlying mechanisms; therefore, an experimental medicine approach and a mechanistic understanding are required to provide solid evidence on which future policies on diet and nutrition for mental health can be based [269]. As no drugs are available to prevent the progression of these neurological disorders, intervention strategies using phytochemicals have been proposed as an alternative form of treatment. Some researchers consider organosulfur compounds as ideal nutraceutical agents as they can serve not only as direct antioxidants trapping electrons, but also have non-antioxidant effects, such as antiplatelet, fibrinolytic, anti-inflammatory, immunomodulatory, anti-aging actions, etc. [270]. Among phytochemicals, isothiocyanate sulforaphane has demonstrated neuroprotective effects in several in vitro and in vivo studies. In particular, the evidence suggests that sulforaphane’s beneficial effects can be mainly ascribed to its peculiar ability to activate the Nrf2/ARE pathway [271]. Sulforaphane has also been shown to stimulate ubiquitin proteasome system (UPS) activity in vitro; moreover, it activates protein degradation machineries (autophagic activities) in both the brain and peripheral tissues [271]. S-allylcysteine (SAC) prevents the progression of AD, and there are multiple mechanisms underlying this ability [272]. Given that Nrf2 is a major switch in the expression of most antioxidant enzymes and PGC-1α, the activation of Nrf2 appears to be a good therapeutic strategy to control oxidative stress in the brain of AD patients. FGF, flavonoids, α-lipoic acid, allicin, and taurine appear to be able to activate the Nrf2-ARE antioxidant defense system [273,274,275,276].

Accumulating data indicate that sulfane sulfur has important functions in cells. The broad diversity of effects suggests that its functions are general and not specific to any tissue or any process. Moreover, it should not be called a “signaling agent” since there is no evidence that it acts in a controlled rise and fall pattern (as with neurotransmitters or hormones) [277]. Both redox sensing and redox signaling use sulfur switches, especially Cys residues in proteins, which are sensitive to reversible oxidation, nitrosylation, glutathionylation, acylation, sulfhydration, or metal binding. Attempts to regulate redox state have focused on electrophiles, which activate potent cellular defense systems against oxidative stress. Nutraceuticals can serve not only as electrophiles, but as a kind of “pro-electrophilic drug” that becomes electrophilic in response to oxidation and then activates the Keap1/Nrf2/ARE transcription pathway to synthesize endogenous antioxidant “phase 2” enzymes [278]. Another important prerequisite of the new concept in redox biology/medicine is the use of compounds that has been termed “pathologically activated therapeutics”, i.e., relatively small molecules are chemically converted to their active form by the very oxidative stress that they are intended to then combat [278].

Summarizing our multifaceted analysis of the role of serotonergic regulation in the pathogenesis of development and treatment options for AD, we come to the generally banal conclusion that the combination of physical activity, positive emotions, and multimodal medications that modulate the serotonergic system is the key to healthy longevity.

## Data Availability

Not applicable.

## Abbreviations

The following abbreviations are used in this manuscript:

5HT	Serotonin
5-HT system	Serotonergic system
AD	Alzheimer’s disease
Aβ	Amyloid β
AβO	Amyloid β oligomers
AC	Adenylate cyclase
ACC	Anterior cingulate cortex
AChE	Acetylcholinesterase
APP	Amyloid precursor protein
sAPPα	Secreted form of APP
AQP4	Aquaporin-4
BChE	Butyrylcholinesterase
BDNF	Brain-derived neurotrophic factor
CNS	Central nervous system
CVS	Cardiovascular system
DRN	Dorsal raphe nucleus
ETC	Electron transport chain
FGF	Fibroblast growth factor
GABA	γ-aminobutyric acid
LTD	Long-term depression
LTP	Long-term potentiation
mAHP	Medium-duration afterhyperpolarization
MDD	Major depressive disorder
mPTP	Mitochondrial permeability transition pore
MRN	Median raphe nucleus
NMDAR	NMDA receptor
Nrf2	Nuclear respiratory factor 2
PANoptosis	Pyroptosis, apoptosis, and necroptosis
PFC	Prefrontal cortex
PNS	Peripheral nervous system
PrPc	Cellular prion protein
pTau	Tau protein
ROS	Reactive oxygen species
SCN	Suprachiasmatic nucleus
SERT	Serotonin transporter
SSRIs	Selective serotonin reuptake inhibitors
TIM	Translocase of the inner membrane
TOM	Translocase of the outer membrane

## Appendix A. Chemical Names for the Research Compounds

5-HTP-DP—N-acetyl-5-hydroxytrytophyl-5-hydroxytryptophan amide.
8-OH-DPAT—7-(Dipropylamino)-5,6,7,8-tetrahydronaphthalen-1-ol.
AS-19—(2S)-5-(1,3,5-Trimethylpyrazol-4-yl)-2-(dimethylamino)tetralin.
BIMU1—33-ethyl-N-[(5S)-8-methyl-8-azabicyclo [3.2.1]octan-3-yl]-2-oxobenzimidazole-1-carboxamide.
BIMU8—N-[(1R,5S)-8-methyl-8-azabicyclo [3.2.1]oct-3-yl]-2-oxo-3-(propan-2-yl)-2,3-dihydro-1H-benzimidazole-1-carboxamide hydrochloride.
BP 554—1-[3-(3,4-methylenedioxyphenoxy)propyl]-4-phenyl-piperazine maleate.
BRL15572—3-(4-(3-chlorophenyl)piperazin-1-yl)-1,1-diphenyl-2-propanol.
BRL54443—5-Hydroxy-3-(1-methylpiperidin-4-yl)-1H-indole.
BW723C86—α-Methyl-5-(2-thienylmethoxy)-1H-indole-3-ethanamine hydrochloride.
CGS 12066B—7-Trifluoromethyl-4-(4-methyl-1-piperazinyl)pyrrolo [1,2-a]-quinoxalinedimaleate.
CP-93129—1,4-Dihydro-3-(1,2,3,6-tetrahydro-4-pyridinyl)-5H-pyrrol [3,2-b]pyridin-5-one dihydrochloride.
DOI—2,5-Dimethoxy-4-iodoamphetamine.
EMD386088—5-Chloro-2-methyl-3-(1,2,3,6-tetrahydro-4-pyridinyl)-1H-indole.
GR113808—[1-[2-[(Methylsulfonyl)amino]ethyl]-4-piperidinyl]methyl 1-methyl-1H-indole-3-carboxylate.
GR-127935—N-[4-methoxy-3-(4-methyl-1-piperazinyl)phenyl]-2′-methyl-4′-(5-methyl-1,2,4-oxadiazol-3-yl)-1-1′-biphenyl-4-carboxamide.
GR 4661—3-[3-(2-Dimethylaminoethyl)-1H-indol-5-yl]-N-(4-methoxybenzyl)acrylamid.
GR55562—3-[3-(Dimethylamino)propyl]-4-hydroxy-N-[4-(4-pyridinyl)phenyl]benzamide dihydrochloride.
L-703,664—2-((3-(2-(dimethylamino)ethyl)-1H-indol-5-yl)methyl)-5-methyl-1,2,5-thiadiazolidine 1,1-dioxide succinate.
LP12—4-(1,1′-Biphenyl)-2-yl-N-(1,2,3,4-tetrahydro-1-naphthalenyl)-1-piperazinehexanamide hydrochloride.
LP44—6-[4-(2-methylsulfanylphenyl)piperazin-1-yl]-N-(1,2,3,4-tetrahydronaphthalen-1-yl)hexanamide hydrochloride.
LP211—4-[1,1′-Biphenyl]-2-yl-N-[(4-cyanophenyl)methyl]-1-piperazinehexanamide.
LY272015—1-[(3,4-dimethoxyphenyl)methyl]-6-methyl-2,3,4,9-tetrahydro-1H-pyrido [3,4-b]indole hydrochloride.
LY310762—1-[2-[4-(4-fluorobenzoyl)piperidin-1-yl]ethyl]-3,3-dimethylindol-2-one hydrochloride.
LY334370—4-fluoro-N-[3-(1-methylpiperidin-4-yl)-1H-indol-5-yl]benzamide.
LY344864—N-[(6R)-6-(dimethylamino)-6,7,8,9-tetrahydro-5H-carbazol-3-yl]-4-fluorobenzamide.
MDL72222—3-tropanyl-3,5-dichlorobenzoat.
MK212—2-chloro-6-piperazin-1-ylpyrazine hydrochloride.
NAN190—1-(2-Methoxyphenyl)-4-(4-phthalimidobutyl)piperazine hydrobromide.
NAS-181—(2R)-2-[[[3-(4-morpholinylmethyl)-2H-1-benzopyran-8-yl]oxy]methyl]-morpholine bimethanesulfonate.
PNU-109291—(S)-3,4-Dihydro-1-[2-[4-(4-methoxyphenyl)-1-piperazinyl]ethyl]-N-methyl-1H-2-benzopyran-6-carboxamide.
R-96544—(2R,4R)-5-[2-[2-[2-(3-Methoxyphenyl)ethyl]phenoxy]ethyl]-1-methyl-3-pyrrolidinol hydrochloride.
Ro 04-6790—4-amino-N-[2,6-bis(methylamino)pyrimidin-4-yl]benzenesulfonamide.
RS100235—1-(5-amino-6-chloro-2,3-dihydro-1,4-benzodioxin-8-yl)-3-[1-[3-(3,4-dimethoxyphenyl)propyl]piperidin-4-yl]propan-1-one.
RS102221—N-[5-[5-(2,4-dioxo-1,3,8-triazaspiro [4.5]decan-8-yl)pentanoyl]-2,4-dimethoxyphenyl]-4-(trifluoromethyl)benzenesulfonamide.
RS127445—4-(4-fluoronaphthalen-1-yl)-6-propan-2-ylpyrimidin-2-amine.
RS67333—1-(4-amino-5-chloro-2-methoxyphenyl)-3-(1-butylpiperidin-4-yl)propan-1-one.
RS67506—N-[2-[4-[3-(4-amino-5-chloro-2-methoxyphenyl)-3-oxopropyl]piperidin-1-yl]ethyl]methanesulfonamide hydrochloride.
SB204070—(1-butylpiperidin-4-yl)methyl 5-amino-6-chloro-2,3-dihydro-1,4-benzodioxine-8-carboxylate.
SB236057—(1′-ethylspiro [6,7-dihydro-2H-furo [2,3-f]indole-3,4′-piperidine]-5-yl)-[4-[2-methyl-4-(5-methyl-1,3,4-oxadiazol-2-yl)phenyl]phenyl]methanone.
SB242084—6-chloro-5-methyl-N-[6-(2-methylpyridin-3-yl)oxypyridin-3-yl]-2,3-dihydroindole-1-carboxamide.
SB258719—(1R)-3,N-dimethyl-N-[1-methyl-3-(4-methylpiperidin-1-yl)propyl]benzenesulfonamide.
SB269970—3-[(2R)-2-[2-(4-methylpiperidin-1-yl)ethyl]pyrrolidin-1-yl]sulfonylphenol.
SB271046—5-chloro-N-(4-methoxy-3-piperazin-1-ylphenyl)-3-methyl-1-benzothiophene-2-sulfonamide.
SB272183—5-chloro-6-(4-methylpiperazin-1-yl)-N-(4-pyridin-4-ylnaphthalen-1-yl)-2,3-dihydroindole-1-carboxamide.
SB399885—N-(3,5-dichloro-2-methoxyphenyl)-4-methoxy-3-piperazin-1-ylbenzenesulfonamide.
SB699551—3-cyclopentyl-N-[2-(dimethylamino)ethyl]-N-[[4-[4-[(2-phenylethylamino)methyl]phenyl]phenyl]methyl]propanamide;dihydrochloride.
SR57227A—1-(6-chloropyridin-2-yl)piperidin-4-amine.
U92016A—(8R)-8-(dipropylamino)-6,7,8,9-tetrahydro-3H-benzo[e]indole-2-carbonitrile
WAY 100635—N-[2-[4-(2-Methoxyphenyl)-1-piperazinyl]ethyl]-N-(2-pyridyl)cyclohexanecarboxamide.
WAY 163909—(11R,15R)-7,10-diazatetracyclo [8.5.1.05,16.011,15]hexadeca-1,3,5(16)-triene.
WAY 181187—2-[1-(6-chloroimidazo [2,1-b][1,3]thiazol-5-yl)sulfonylindol-3-yl]ethanamine.
